# Supplementary material for: HIVprotI: an integrated web based platform for prediction and design of HIV proteins inhibitors
Source: J Cheminform. 2018 Mar 9;10:12. doi: 10.1186/s13321-018-0266-y (PMC5845081; doi:10.1186/s13321-018-0266-y)
Supplement: Supplementary file 2 — Additional file 2. Source code of HIVProtI web server. [file 13321_2018_266_MOESM2_ESM.zip › HIVprotI_Source-code/hivprotI/weka-3-6-6/doc/weka/classifiers/functions/neural/NeuralConnection.html]

NeuralConnection


---


|  |  |  |
| --- | --- | --- |
| **Overview** \| **Package** \| **Class** \| **Tree** \| **Deprecated** \| **Index** \| **Help** \| **Weka's home** | |  |
| **PREV CLASS**   **NEXT CLASS** | **FRAMES**    **NO FRAMES**     **All Classes** |  |
| SUMMARY: NESTED \| FIELD \| CONSTR \| METHOD | DETAIL: FIELD \| CONSTR \| METHOD |  |


---


## weka.classifiers.functions.neural Class NeuralConnection

```
java.lang.Object
  weka.classifiers.functions.neural.NeuralConnection
```

**All Implemented Interfaces:**: java.io.Serializable, RevisionHandler

**Direct Known Subclasses:**: NeuralNode

---

``` public abstract class NeuralConnection extends java.lang.Object implements java.io.Serializable, RevisionHandler ```

Abstract unit in a NeuralNetwork.

**Version:**
:   $Revision: 5403 $

**Author:**
:   Malcolm Ware (mfw4@cs.waikato.ac.nz)

**See Also:**: Serialized Form

---

| **Field Summary** | |
| --- | --- |
| `static int` | `CONNECTED`             This flag is set once the unit has a connection. |
| `static int` | `INPUT`             This unit is an input unit. |
| `static int` | `OUTPUT`             This unit is an output unit. |
| `static int` | `PURE_INPUT`             This unit is a pure input unit. |
| `static int` | `PURE_OUTPUT`             This unit is a pure output unit. |
| `static int` | `UNCONNECTED`             This unit is not connected to any others. |


**Constructor Summary**

`NeuralConnection(java.lang.String id)`
  
          Constructs The unit with the basic connection information prepared for
use.


| **Method Summary** | |
| --- | --- |
| `static boolean` | `connect(NeuralConnection s, NeuralConnection t)`             Connects two units together. |
| `static boolean` | `disconnect(NeuralConnection s, NeuralConnection t)`             Disconnects two units. |
| `void` | `drawHighlight(java.awt.Graphics g, int w, int h)`             Call this function to draw the node highlighted. |
| `void` | `drawInputLines(java.awt.Graphics g, int w, int h)`             Call this function to draw the nodes input connections. |
| `void` | `drawNode(java.awt.Graphics g, int w, int h)`             Call this function to draw the node. |
| `void` | `drawOutputLines(java.awt.Graphics g, int w, int h)`             Call this function to draw the nodes output connections. |
| `abstract  double` | `errorValue(boolean calculate)`             Call this to get the error value of this unit. |
| `java.lang.String` | `getId()` |
| `int[]` | `getInputNums()`             Use this to get easy access to the input numbers. |
| `NeuralConnection[]` | `getInputs()`             Use this to get easy access to the inputs. |
| `int` | `getNumInputs()` |
| `int` | `getNumOutputs()` |
| `int[]` | `getOutputNums()`             Use this to get easy access to the output numbers. |
| `NeuralConnection[]` | `getOutputs()`             Use this to get easy access to the outputs. |
| `int` | `getType()` |
| `double` | `getX()` |
| `double` | `getY()` |
| `boolean` | `onUnit(java.awt.Graphics g, int x, int y, int w, int h)`             Call this function to determine if the point at x,y is on the unit. |
| `abstract  double` | `outputValue(boolean calculate)`             Call this to get the output value of this unit. |
| `void` | `removeAllInputs()`             This function will remove all the inputs to this unit. |
| `void` | `removeAllOutputs()`             This function will remove all outputs to this unit. |
| `abstract  void` | `reset()`             Call this to reset the unit for another run. |
| `abstract  void` | `restoreWeights()`             Call this to have the connection restore from the saved weights. |
| `abstract  void` | `saveWeights()`             Call this to have the connection save the current weights. |
| `void` | `setType(int t)` |
| `void` | `setX(double x)` |
| `void` | `setY(double y)` |
| `void` | `updateWeights(double l, double m)`             Call this function to update the weight values at this unit. |
| `double` | `weightValue(int n)`             Call this to get the weight value on a particular connection. |

**Methods inherited from class java.lang.Object**

`equals, getClass, hashCode, notify, notifyAll, toString, wait, wait, wait`

**Methods inherited from interface weka.core.RevisionHandler**

`getRevision`

**Field Detail**

### UNCONNECTED

```
public static final int UNCONNECTED
```

:   This unit is not connected to any others.

    **See Also:**: Constant Field Values

---


### PURE\_INPUT

```
public static final int PURE_INPUT
```

:   This unit is a pure input unit.

    **See Also:**: Constant Field Values

---


### PURE\_OUTPUT

```
public static final int PURE_OUTPUT
```

:   This unit is a pure output unit.

    **See Also:**: Constant Field Values

---


### INPUT

```
public static final int INPUT
```

:   This unit is an input unit.

    **See Also:**: Constant Field Values

---


### OUTPUT

```
public static final int OUTPUT
```

:   This unit is an output unit.

    **See Also:**: Constant Field Values

---


### CONNECTED

```
public static final int CONNECTED
```

:   This flag is set once the unit has a connection.

    **See Also:**: Constant Field Values


**Constructor Detail**

### NeuralConnection

```
public NeuralConnection(java.lang.String id)
```

:   Constructs The unit with the basic connection information prepared for
    use.

    **Parameters:**: `id` - the unique id of the unit


**Method Detail**

### getId

```
public java.lang.String getId()
```

:   **Returns:**: The identity string of this unit.

---


### getType

```
public int getType()
```

:   **Returns:**: The type of this unit.

---


### setType

```
public void setType(int t)
```

:   **Parameters:**: `t` - The new type of this unit.

---


### reset

```
public abstract void reset()
```

:   Call this to reset the unit for another run.
    It is expected by that this unit will call the reset functions of all
    input units to it. It is also expected that this will not be done
    if the unit has already been reset (or atleast appears to be).

---


### outputValue

```
public abstract double outputValue(boolean calculate)
```

:   Call this to get the output value of this unit.

    :   **Parameters:**: `calculate` - True if the value should be calculated if it hasn't been already. **Returns:**: The output value, or NaN, if the value has not been calculated.

---


### errorValue

```
public abstract double errorValue(boolean calculate)
```

:   Call this to get the error value of this unit.

    :   **Parameters:**: `calculate` - True if the value should be calculated if it hasn't been already. **Returns:**: The error value, or NaN, if the value has not been calculated.

---


### saveWeights

```
public abstract void saveWeights()
```

:   Call this to have the connection save the current
    weights.

---


### restoreWeights

```
public abstract void restoreWeights()
```

:   Call this to have the connection restore from the saved
    weights.

---


### weightValue

```
public double weightValue(int n)
```

:   Call this to get the weight value on a particular connection.

    :   **Parameters:**: `n` - The connection number to get the weight for, -1 if The threshold weight should be returned. **Returns:**: This function will default to return 1. If overridden, it should return the value for the specified connection or if -1 then it should return the threshold value. If no value exists for the specified connection, NaN will be returned.

---


### updateWeights

```
public void updateWeights(double l,
                          double m)
```

:   Call this function to update the weight values at this unit.
    After the weights have been updated at this unit, All the
    input connections will then be called from this to have their
    weights updated.

    :   **Parameters:**: `l` - The learning Rate to use.: `m` - The momentum to use.

---


### getInputs

```
public NeuralConnection[] getInputs()
```

:   Use this to get easy access to the inputs.
    It is not advised to change the entries in this list
    (use the connecting and disconnecting functions to do that)

    :   **Returns:**: The inputs list.

---


### getOutputs

```
public NeuralConnection[] getOutputs()
```

:   Use this to get easy access to the outputs.
    It is not advised to change the entries in this list
    (use the connecting and disconnecting functions to do that)

    :   **Returns:**: The outputs list.

---


### getInputNums

```
public int[] getInputNums()
```

:   Use this to get easy access to the input numbers.
    It is not advised to change the entries in this list
    (use the connecting and disconnecting functions to do that)

    :   **Returns:**: The input nums list.

---


### getOutputNums

```
public int[] getOutputNums()
```

:   Use this to get easy access to the output numbers.
    It is not advised to change the entries in this list
    (use the connecting and disconnecting functions to do that)

    :   **Returns:**: The outputs list.

---


### getX

```
public double getX()
```

:   **Returns:**: the x coord.

---


### getY

```
public double getY()
```

:   **Returns:**: the y coord.

---


### setX

```
public void setX(double x)
```

:   **Parameters:**: `x` - The new value for it's x pos.

---


### setY

```
public void setY(double y)
```

:   **Parameters:**: `y` - The new value for it's y pos.

---


### onUnit

```
public boolean onUnit(java.awt.Graphics g,
                      int x,
                      int y,
                      int w,
                      int h)
```

:   Call this function to determine if the point at x,y is on the unit.

    :   **Parameters:**: `g` - The graphics context for font size info.: `x` - The x coord.: `y` - The y coord.: `w` - The width of the display.: `h` - The height of the display. **Returns:**: True if the point is on the unit, false otherwise.

---


### drawNode

```
public void drawNode(java.awt.Graphics g,
                     int w,
                     int h)
```

:   Call this function to draw the node.

    :   **Parameters:**: `g` - The graphics context.: `w` - The width of the drawing area.: `h` - The height of the drawing area.

---


### drawHighlight

```
public void drawHighlight(java.awt.Graphics g,
                          int w,
                          int h)
```

:   Call this function to draw the node highlighted.

    :   **Parameters:**: `g` - The graphics context.: `w` - The width of the drawing area.: `h` - The height of the drawing area.

---


### drawInputLines

```
public void drawInputLines(java.awt.Graphics g,
                           int w,
                           int h)
```

:   Call this function to draw the nodes input connections.

    :   **Parameters:**: `g` - The graphics context.: `w` - The width of the drawing area.: `h` - The height of the drawing area.

---


### drawOutputLines

```
public void drawOutputLines(java.awt.Graphics g,
                            int w,
                            int h)
```

:   Call this function to draw the nodes output connections.

    :   **Parameters:**: `g` - The graphics context.: `w` - The width of the drawing area.: `h` - The height of the drawing area.

---


### removeAllInputs

```
public void removeAllInputs()
```

:   This function will remove all the inputs to this unit.
    In doing so it will also terminate the connections at the other end.

---


### removeAllOutputs

```
public void removeAllOutputs()
```

:   This function will remove all outputs to this unit.
    In doing so it will also terminate the connections at the other end.

---


### getNumInputs

```
public int getNumInputs()
```

:   **Returns:**: The number of input connections.

---


### getNumOutputs

```
public int getNumOutputs()
```

:   **Returns:**: The number of output connections.

---


### connect

```
public static boolean connect(NeuralConnection s,
                              NeuralConnection t)
```

:   Connects two units together.

    :   **Parameters:**: `s` - The source unit.: `t` - The target unit. **Returns:**: True if the units were connected, false otherwise.

---


### disconnect

```
public static boolean disconnect(NeuralConnection s,
                                 NeuralConnection t)
```

:   Disconnects two units.

    :   **Parameters:**: `s` - The source unit.: `t` - The target unit. **Returns:**: True if the units were disconnected, false if they weren't (probably due to there being no connection).


---


|  |  |  |
| --- | --- | --- |
| **Overview** \| **Package** \| **Class** \| **Tree** \| **Deprecated** \| **Index** \| **Help** \| **Weka's home** | |  |
| **PREV CLASS**   **NEXT CLASS** | **FRAMES**    **NO FRAMES**     **All Classes** |  |
| SUMMARY: NESTED \| FIELD \| CONSTR \| METHOD | DETAIL: FIELD \| CONSTR \| METHOD |  |


---
